# Rapid kinetics of AAV8 delivery of Vglut3 and hearing rescue shown day-by-day via matched IHC and ABR

**DOI:** 10.1016/j.omtm.2025.101638

**Published:** 2025-11-27

**Authors:** Cole W.D. Peters, Killian Hanlon

**Affiliations:** 1 Division of Pediatric Hematology-Oncology, Department of Pediatrics, University of California, Los Angeles, Los Angeles, CA, USA; 2 Lir Therapeutics, London, UK

## Main text

The race to implement effective gene therapy to restore hearing and sight has relied primarily on adeno-associated virus (AAV)-based vectors, due to their wide range of cell tropisms, ease of modification, and their relatively innocuous safety profile in patients. Delivery to the inner ear is non-trivial; researchers have developed multiple injection routes to reach the cochlea in animal models, which is nestled deep within the temporal bone of the skull. Newborn mice developmentally resemble a human fetus in the final month of gestation, with the eyelids and ears not fully developed. This allows researchers to inject AAV into the cochlea via the round window membrane—a straightforward procedure that is widely adopted and relatively reproducible. Around a week after birth in mice, this becomes near impossible as the round window niche becomes a bony structure. This same phenomenon occurs in humans during their 16th week as a fetus, which supports the model of newborn mice as applicable to what *in utero* gene therapy may provide for human patients. However, for disease interventions in children and adults, assessing other routes of administration is critical.

In a recent issue of *Molecular Therapy Methods and Clinical Development*, the authors demonstrate that delivery of an AAV vector restores expression of Vglut3 and restoration of hearing in 4- to 5-week-old adult mice^1^ and demonstrate the rapid kinetics of transgene expression through matched auditory brain stem response (ABR) hearing tests and immunohistochemistry of the inner and outer hair cells of the cochlea. While research into Vglut3 gene therapy for murine deafness has been performed before, the authors of this paper precisely demonstrate the onset of Vglut3 expression from day 1 through the 2nd week after delivery, paired with hearing tests to directly compare growing Vglut3 expression to rescue of hearing in adult Vglut3 knockout mice.

Vesicular glutamate transporter 3 (VGLUT3) is encoded by the SLC17A8 gene, mutations in which can cause autosomal dominant nonsyndromic deafness, DFNA25.^2^ To address this gene deficit, Zhao and co-authors deliver an AAV8 capsid carrying a CMV-Vglut3-FLAG construct via canalstomy to the posterior semicircular canal to 5-week-old mice carrying a knockout mutation. This work closely resembles a previous article by Zhao et al.,^3^ in which they delivered the same virus to adult mice and observed long-term (12–16 weeks) restoration of hearing beginning as early as 2 weeks post delivery. Here, Zhao and colleagues go further, with a day-by-day study measuring hearing and Vglut3 expression each day post AAV delivery. They find that as early as day 3 post injection, Vglut3 expression appears, and statistically significant increases in expression are observed by day 5. Paired with this are ABRs from the mice, which demonstrate significant improvement in middle-tone (11–16 kHz) detection after 1 day, with hearing in the low/middle and high frequencies restored to wild-type levels by day 3 and 5 post injection, respectively.

The novelty of this work comes from the demonstration that unmodified AAV8 with a simple CMV promoter-driven cDNA—delivered at a moderate dose (2–3 × 10^10^ vg)—was capable of solely transducing inner hair cells and spurred quick rescue of hearing in adult mice. Usually, capsid formation and transgene promoter selection are the focus of ways to improve gene therapy delivery for most deafness and blindness studies. However, the delivery method, canalostomy through the posterior semi-circular canal, is more challenging than methods such as cochleostomy or round window delivery.^4^^,^^5^

**Figure 1:**
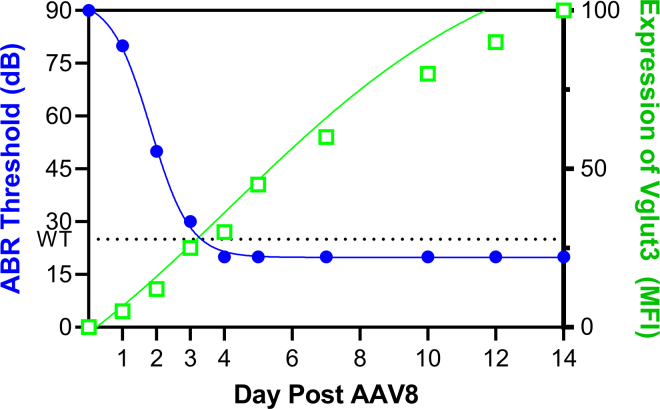
Zhao et al. demonstrate that ABR threshold quickly drops to wild-type levels, restoring hearing, after 1 to 3 days of growing Vglut3 transgene expression

What is unique about this paper is the demonstration that Vglut3 transgene expression is able to restore hearing capacity in such a rapid manner in adult mice (Figure 1). There are several implications which may serve the deafness gene therapy research community. First and foremost is the rapidity of hearing restoration. Only 1 day post injection is needed to observe a material drop in ABR thresholds in the middle-tone regions. This argues that protein expression in inner hair cells is robustly dynamic, and delivery of a constituent promoter is able to quickly provide the absent protein being targeted in a functional way to the inner hair cells. Here, the authors are rescuing production of a synaptic transport protein, which may have less post-translational modifications or easier transport to its required intracellular niche than other deafness-related proteins. The transport and modification of an extracellular deafness-associated protein-like protocadherin-15 or Cadherin-23 could require more time, but that has yet to be proven. Secondly, the amount of protein required to functionally rescue hearing is often raised in questions about gene therapy efficacy. Here, the authors demonstrate that even weakly stained inner hair cells (IHCs), at days 1 through 3, were potent enough to restore functional hearing. Additionally, the authors demonstrate that as Vglut3 expression increases, the ABR threshold drops. Finally, this article’s demonstration that the canalstomy delivers reliable transgene expression with AAV should be a signal for laboratories to become acquainted with this procedure for use in adult mice models.

The broader impact this article has for the field of gene therapy is less well known. While VGLUT3 mutations do cause deafness in humans, there are no clinical trials for patients, and the only study carried out in the United States to determine incidence was terminated due to “No convincing results” (NCT01802190). This is often cited as a weakness for researchers studying this deafness as there are no directly translatable diseases to which breakthroughs like this can be applied, and the authors point this out in their own discussion. Indeed, Vglut3 deficiency still allows IHC and outer hair cell (OHC) development. This is different from other deafness mutations that completely ablate IHC or OHC architecture and therefore may require *in utero* treatment to rescue. Additionally, the mouse model used (a recessive knockout) may not accurately mimic the pathology of human DFNA25. However, in conjunction with Dr. Zhao’s earlier work in 2022, which showed that Vglut3 rescue begins to wane after 22 weeks, this study presents an elegant timeline for AAV8 delivery to adult mice, the kinetics of transgene expression, and demonstrates a direct link between transgene expression level and restoration of hearing. Taken together, groups researching adult onset deafness should use this article as a solid methodological foundation for their studies.

## Declaration of interests

C.W.D.P. is an associate project scientist at the University of California, Los Angeles. K.H. is a director of Lir Therapeutics Limited, a UK-based gene therapy company.
